# Expanding the employment quality framework: Health and precarity in the lives of black emerging adults

**DOI:** 10.1016/j.ssmqr.2026.100743

**Published:** 2026-03-18

**Authors:** Holly Nishimura, Sarah Andrea, Michelle Nakphong, Darius Jovon Bright, Alexandria Downing, Roddrick Dugger, Margaret Libby, Marguerita Lightfoot, Sheri A. Lippman, Emily Arnold

**Affiliations:** a Department of Medicine, University of California San Francisco, San Francisco, CA, USA; b School of Public Health, Oregon Health and Science University, Portland, OR, USA; c MyPath, San Francisco, CA, USA

**Keywords:** Precarious employment, Employment quality, Black emerging adults, Health inequities, Qualitative research, Mental health

## Abstract

Precarious employment, a condition marked by job instability, low wages, and limited protections, exacerbates economic insecurity and poor health, yet little is known about its impact on Black emerging adults (BEA). This study draws on longitudinal qualitative data to examine how young adults ages 18–24 experience and navigate precarious work while participating in a guaranteed income trial. We conducted in-depth interviews with 36 participants at baseline and 31 at 12-months follow-up. Transcripts were coded and then we conducted framework analysis of excerpts related to employment, health, and healthcare seeking. Four dimensions of employment precarity were salient in participants' accounts: employment instability, unpredictable schedules and time scarcity, unsafe and unsupportive work environments, and constrained agency. Time scarcity was driven by inconsistent hours, multiple jobs, caregiving, and long commutes; it limited participants’ ability to rest, pursue education, and access health care. Although participants demonstrated agency by making strategic decisions about work, school, and caregiving, their choices were constrained by immediate financial pressures. Employment precarity persisted over time, with overlapping experiences of low wages, unpredictable scheduling, and unsafe conditions undermining both physical and mental health. These findings highlight how precarious employment was experienced as a structural constraint on health during emerging adulthood, forcing difficult tradeoffs that compound existing inequities. While participants receiving guaranteed income during the study period sometimes described the cash transfers as easing short-term financial pressure, structural interventions such as stronger workplace protections, accessible childcare, and workforce development are urgently needed to disrupt cycles of precarity and improve health outcomes.

## 1. Introduction

The unequal distribution of money, power, and resources shapes the conditions in which people live and work and is a fundamental determinant of health (Kawachi & Subramanian, 2018). Attention has increasingly focused on employment quality as a central pathway through which these broader structural inequalities become embodied. Employment quality is defined as a multidimensional construct encompassing the full continuum of employment conditions that result from power relations between workers and employers. A growing body of evidence links low employment quality, known as precarious employment, to adverse outcomes including psychological distress, injury, and chronic illness (Hajat et al., 2024; Pulford et al., 2022). Precarious employment is often characterized by temporary contracts, unstable work time arrangements, insufficient wages, limited autonomy, and a lack of social protections (Hajat et al., 2024; Julia et al., 2017; Kreshpaj et al., 2020). In the United States, the prevalence of precarious employment has increased substantially in recent decades, with the burden falling disproportionately on women, racially minoritized groups, and individuals with lower levels of formal education (Oddo et al., 2021). As such, employment quality has emerged as a key social determinant of health and an important mechanism through which structural inequality is reproduced.

Emerging adulthood, the critical life stage from age 18 to 29, is when many individuals enter the workforce and establish their early employment experiences. These early labor market experiences are particularly consequential because they occur at a time when educational trajectories, caregiving responsibilities, and economic independence are still in flux. This period serves as a key phase for the reproduction or disruption of health and socioeconomic inequities over the life course (Arnett et al., 2014; Barlett et al., 2020). The quality of early employment experiences can have lasting effects on health, earnings, and educational attainment. As a structural driver of socioeconomic status, employment quality can serve as a turning point through which early exposure to low-quality employment conditions can constrain future life planning, increase stress, and reinforce existing inequities. Conversely, access to high-quality employment with adequate wages, flexibility for continued education, and social protections can promote stability and resilience, providing a pathway to upward economic mobility (Kalleberg, 2011; Oreopoulos et al., 2012; Western et al., 2012).

Despite the importance of early employment experiences for life-course trajectories, the experiences of emerging adults in the U.S. remain critically underexamined. Much of the existing literature on precarious employment has focused on mid-career adults (Andrea et al., 2022; Eisenberg-Guyot et al., 2020; Patil et al., 2020), often in European settings with stronger labor protections and social safety nets (Benach et al., 2014; Vanroelen, 2019). U.S.-based qualitative research has documented how low-wage and precarious work is experienced as chronic insecurity, time scarcity, and constrained choice, with workers frequently normalizing poor conditions while relying on individual strategies to manage economic stress (Edin & Shaefer, 2015; Henly et al., 2006; Newman, 1999). However, this body of work has largely focused on occupational sectors such as gig work, caregiving, retail, or food service (Keke, 2013; Zoeckler, 2018), offering limited insight into how precarious employment shapes early labor market participation and health during emerging adulthood.

Precarious employment may be particularly consequential for emerging adults facing structural disadvantage, including Black Americans, who are overrepresented in low-wage and unstable jobs due to a history of structural racism, economic exclusion, and discriminatory policy (Wilson, 1996). Racialized labor market segmentation and unequal access to educational and employment opportunities shape not only the types of jobs available to Black emerging adults (BEA), but also the risks those jobs pose to health (Bailey et al., 2017). Understanding precarity and its consequences among young, racially minoritized workers requires examining both the structures that constrain opportunities and the individual agency (the capacity to make decisions and act within constrained environments) through which individuals seek control and mobility (Emirbayer & Mische, 1998). For low-income=BEA, navigating precarious employment often involves balancing immediate survival with longer-term goals such as education, training, caregiving, or greater stability (Silva, 2013). These tradeoffs are shaped by time scarcity, financial insecurity, and limited institutional support, yet remains insufficiently integrated into employment quality frameworks.

In particular, less is known about how employment precarity intersect in early employment experiences to shape health. To address these gaps, this study examines the employment experiences of low-income BEA enrolled in the Black Economic Equity Movement (BEEM), a randomized trial of guaranteed income. Using in-depth interviews collected at two time points, we explore how participants navigate employment precarity, how these dynamics affect their health, and how agency operates within structural constraints. This research serves to extend our understanding of the employment quality framework in a structurally marginalized young adult population in the United States.

## 2. Methods

### 2.1. Study design and parent study (BEEM)

The data for this study come from a nested longitudinal cohort within BEEM, a randomized controlled crossover trial. BEEM tested the impact of guaranteed income on mental health, financial wellbeing, and health services utilization among 300 low-income BEA ages 18–24 residing in Oakland and San Francisco, California. Low-income status was determined using residential location rather than self-reported income. Eligibility required residence in a LIHTC Qualified Census Tract in San Francisco or Oakland, or verified unhoused status within either city, consistent with the BEEM trial protocol (Lippman et al., 2023). Participants were randomized to one of two study arms: the “Cash Now” arm received $500 per month during the first 12-months of follow-up, and the “Cash Later” arm received $500 per month during the second 12-months, totaling of 24-months of follow-up. All participants were offered optional financial mentoring to bolster financial capability. The BEEM trial protocol is described in detail elsewhere (Lippman et al., 2023).

### 2.2. Recruitment and sampling

For the nested qualitative study, we purposively sampled for variation in age (18–20 vs 21–24), city of residence (Oakland vs San Francisco), gender identity (male, female, transgender/non-binary), and study arm (Cash Now vs Cash Later). At enrollment, all BEEM participants were asked about their interest in participating in qualitative interviews. Participants who expressed interest were contacted for enrollment in the qualitative study by qualitative investigators. Among those interested, the team also monitored enrollment to avoid excluding participants who were parenting or expressed interest in mentoring. A total of 36 participants were enrolled in the qualitative cohort. Although we did not purposively sample on characteristics such as housing status, educational enrollment, caregiving responsibilities, or participation in the optional financial mentoring program, these factors emerged as salient influences on participants’ employment experiences and health. We therefore describe these characteristics descriptively in the sample to provide contextual information relevant to interpretation of the findings.

### 2.3. Data collection and interviewer positionality

Participants were interviewed using semi-structured guides at two time points: baseline (approximately one month after BEEM enrollment) and 12-months follow-up. In-depth interviews (IDIs) were conducted remotely over Zoom, lasted between 45 and 60 min, were recorded, transcribed, and participants were compensated $40 for each interview.

BEEM participants completed baseline IDIs between November 2022 and August 2023. Baseline IDIs covered a broad range of topics including day-to-day life, housing, employment, social support, financial status, physical and mental health, healthcare utilization and access, and community engagement. Interview questions included: “Tell me a little bit about your day-to-day life.“; “Tell me a little bit about how you make money.“; “What kinds of things do you do to support yourself?“. The same questions were asked in the 12-month follow-up interviews.

Interviews were conducted by three trained qualitative interviewers (DJB, HN, MN). HN and MN are PhD-level trained social scientists and Asian American women with expertise in social determinants of health and qualitative methods. DJB is a community-based researcher and African American man with extensive experience conducting interviews with marginalized communities in the San Francisco Bay Area. All interviewers were trained in the study's protocol, ethical considerations, and use of the semi-structured interview guide. Regular debriefs were held to review interviewing strategies, share insights, and refine approaches for follow-up interviews.

This study, consent forms, and all study procedures were reviewed and approved by the University of California, San Francisco Institutional Review Board (IRB #:21–35420). All participants provided written consent to participate in the BEEM study and an additional verbal consent to participate in the qualitative interviews.

### 2.4. Data analysis

Data analysis began with early coding and analytic discussions among the qualitative team, with interviewers bringing insights from their experiences conducting interviews and other members providing contextual expertise. The qualitative team developed an initial codebook based on interview guide questions and additional codes were added through an inductive process after close reading of the transcripts. To ensure reliability and consensus, the team refined the codebook iteratively by independently coding and discussing code definitions and application on three distinct IDI transcripts (one transcript by each of the three interviewers), finalizing the codebook and establishing well defined definitions of parent and child codes. Primary and secondary coding of each transcript was completed in Dedoose (SocioCultural Research Consultants, 2023) and analysts met to discuss and resolve any coding discrepancies. Relevant codes for this analysis included: “descriptions of employment; ” “other income sources; ” “financial status; ” “financial issue/challenge; ” “mental health; ” “physical health; ” “sexual and reproductive health; ” health insurance; ” “unmet health need.”

We used a framework analysis approach to systematically organize and compare participant experiences across the two time points (Gale et al., 2013). This method is well suited for applied research, allowing for structured analysis informed by existing theory while maintaining flexibility to incorporate emerging themes. In contrast to a purely inductive thematic analysis, framework analysis involved the development of an analytic matrix that enabled systematic comparison across participants and across time, facilitating longitudinal and cross-case analysis. Deductive codes were informed by the existing literature on precarious employment and health disparities (Kreshpaj et al., 2020). Specifically, we used the three major domains of precarious employment identified by Kreshpaj et al.: employment insecurity, income inadequacy, lack of rights and protection. Inductive coding captured new, emergent themes from the data such as perceived time scarcity, the emotional toll of instability, and individual agency in navigating limited work opportunities. Data were systematically abstracted and organized into a matrix allowing for comparison across participants and time points. Special attention was given to how participants' experiences evolved including participants’ descriptions of how guaranteed income and other supports related to their experiences. Given changes over the 12 months, a longitudinal comparison was conducted to capture shifts in employment status, perceptions of job security, financial stress, and health. Patterns in employment quality were examined, along with contextual factors (e.g., caregiving responsibilities, housing instability, educational enrollment, and access to social support) influencing these trajectories. To support analytic rigor and interpretation, findings were discussed iteratively within the research team, including community-based investigators, and were informed by ongoing reflexive discussion and informal member checking during follow-up interviews.

## 3. Results

Characteristics of study participants at baseline and 12-months follow-up are shown in Table 1. We selected 19 participants from the Cash Now study arm and 17 participants from the Cash Later arm for participation in the qualitative cohort. Thirty-one individuals participated in 12-month follow-up interviews (86% retention). Reasons for attrition at 12-months follow-up included death (n = 1), unresponsive to follow-up attempts (n = 3), and declining to participate in the second interview following a traumatic event (n = 1).

Participants described precarious employment as a set of overlapping challenges including unstable income, unpredictable schedules, unsafe work conditions, and limited protections. These experiences were dynamic, often compounded over time, and fundamentally influenced their daily routines, health, and long-term goals. Throughout the results, references to how precarious employment shapes or affects health reflect participants’ perceived connections between work conditions and health. We identified four interrelated themes that capture the experience of employment precarity among BEA: (1) employment instability; (2) unpredictable schedules and time scarcity; (3) unsafe and unsupported work environments; and (4) constrained agency. We then present a cross-cutting section (3.5) describing how participants linked these employment conditions with health and healthcare access. The following sections explore each of these themes.

### 3.1. Employment instability

Participants described employment instability as a defining feature of their work lives, characterized by tenuous and uncertain arrangements rather than stable or predictable employment. Many were employed through temporary contracts, third-party staffing agencies, or short-term trainee programs that offered no guarantee of continued work. These arrangements left participants in a persistent state of uncertainty, with some describing feeling “replaceable” within their workplaces. Across accounts, employment instability limited opportunities for advancement and made it difficult to plan consistently from one pay period to the next.

**Low and inconsistent wages** compounded this instability, creating ongoing financial strain and material deprivation. To meet basic needs with the high cost of living in the San Francisco Bay Area, including Oakland, many worked multiple jobs or picked up extra shifts whenever possible. Security guard contracts were especially common, with several participants holding more than one position or picking up additional shifts to make ends meet. One woman working two security jobs explained that nearly all her waking hours were spent on the job:

“Well, in my day-to-day life, I work very often. Even on my off days, I work. Yeah. There's nothing really much to it. […] I just feel like I just got to stay ahead of bills and stuff like that because it's really expensive to live in the Bay Area. So, I just try to stay ahead, and I just call in to get extra shifts.”

- IDI 30, age 21–24, Female, Oakland, Cash Later

In addition to working multiple jobs, participants described engaging in **supplementary income-generating activities** like gig work (e.g., meal delivery), hair dressing, babysitting, and trimming marijuana to meet their basic needs. The necessity to generate supplementary income was frequently discussed as physically and emotionally taxing. While health consequences varied, participants described fatigue, strain, and challenges managing existing health conditions in the context of ongoing economic insecurity. For example, one participant described experiencing anemia following the birth of her son while also discussing plasma donation as a strategy for managing financial precarity:

“I got it [anemia] after I had my son. That was tough. They were like, ‘Oh, yeah. You're kind of anemic now.’ I don't know why. And then, I tested again. And they're like, “Yeah, you're still anemic.” I thought it would go away, but apparently not. So I take iron pills every once in a blue moon … Yeah. Because I don’t always have enough iron in the stuff that I eat. Because I forget which ones contain iron. So, sometimes, I have to remind myself, like, “Oh. You got to get this in your body.” And, if not, like it’ll be super cold, to the point where I’m like shaking like a dog, pretty much, like a wet dog or something.”

- IDI 4, age 18–20, Female, Oakland, Cash Now, parent

Taken together, participants underscored the pervasive nature of employment instability, as they assembled low-wage and precarious work arrangements often supplemented by informal or gig-based income, threatening both economic security and health.

### 3.2. Unpredictable schedules and time scarcity

A second critical dimension of precarity was **time-related scarcity**, characterized by limited control over when and how time is allocated due to unpredictable schedules, insufficient or variable hours, and the need to combine multiple jobs. Participants did not describe schedule unpredictability and multiple job holding as separate phenomena; rather, unstable schedules and inadequate hours often necessitated juggling additional work, intensifying time scarcity and constraining agency over daily routines. Participants working in fast food, retail, and afterschool programs commonly described part-time and variable shifts with little advance notice, sometimes receiving schedules the night before or day of a shift. This instability made it difficult to plan for childcare, school, or additional income-generating work. Despite a desire to work more hours, many were unable to secure consistent or adequate schedules, reinforcing ongoing uncertainty about income and time use.

Frustrations with unpredictable scheduling and **limited power to control working hours** led some participants to voluntarily leave jobs, even without having secured new employment. Some participants linked these dynamics to **inability to plan ahead** which participants described as stressful and difficult to manage:

“I was working at [fast food chain] until December. Their managing is terrible. So, I just decided that that wasn’t the place for me. I was barely getting put on schedule and stuff like that. So, there's no point in working somewhere where they barely put you on schedule.”

- IDI 25, age 18–20, Male, Oakland, Cash Later

“With my jobs, I can never count on my hours staying the same. Sometimes they cut hours because they have so many people working, and I don’t understand why they do that. It’s like, ‘What? That doesn’t make any sense.’ One week, I think I’m doing okay, and the next week, my paycheck is short, and I’m struggling again.”

- IDI 21, age 18–20, Female, San Francisco, Cash Later

Beyond income, scheduling precarity constrained participants’ ability to establish stable daily routines and plan time outside of work. One young parent described how unpredictable fast food shifts disrupted routines and left little room for stability:

“Usually, I work evenings and nights. So I wake up kind of late sometimes, depending on what my shift looks like. Like, yesterday, I worked 12:30pm to 4:00pm. So that was kind of different for me. So it was earlier to do everything I usually do. But, since it is a fast food job, it just fluctuates and changes.”

- IDI 4, age 18–20, Female, Oakland, Cash Now, parent

For many participants, unpredictable or insufficient hours in a primary job necessitated taking on additional work, intensifying time scarcity and limiting control over non-work activities. One woman, working two security jobs, contributed to household expenses while saving for college. She described the burden of juggling financial obligations and long work hours with her goal of eventually pursuing higher education:

“Yeah, that [having to work multiple jobs to cover bills] is the thing. That's the only thing that's, like, slowing me down [from going to school]. That would be, like, the only thing, but I don't. It seems like now, you kind of gotta, like, kind of have either a job that's paying about probably like $30 an hour or so, or like two jobs because where I stay at, it's like I'm helping my mom with bills and stuff because it is high. […] I've been putting money aside from my paychecks and then just try to save it to see how much I'll be able to have by the end of this year.”

- IDI 21, age 18–20, Female, San Francisco, Cash Later

Another participant explained that they no longer had time to go to church, an important activity they did with their girlfriend and a method they used for dealing with stress.

“… I'm trying to get another job right now so I can get all of that. Because I get off at 2:00 here, so I’ve got time. I’ve got a lot of that window time. As long as it's in this area though. As long as I don't have to travel anywhere, I'll be able to do a whole other eight hours somewhere else. […] I'll probably add, like 20 [hours per week], so I could do some days, and then have some days for rest days. Shoot, push comes to shove, I'll do it on the weekends because I don’t but the only thing with the weekends is, I go to church every Sunday. I just got baptized on the 4th with my girlfriend. So we don't really open our Sundays for anything anymore.”

- IDI 11, age 18–20, Non-Binary, San Francisco, Cash Now

In contrast, a minority of participants described positions that allowed **agency over their schedules** and location of work (home vs. on site). Having a high level of agency was viewed positively, particularly for parents. One participant, for example, described valuing the autonomy to structure his workday around parenting responsibilities and to complete tasks independently and without supervision:

“Whenever I was done with my kids, dropping them off at school, my supervisor knew I would go there and get the job done. And it was individual work, so there’s nobody I checked into. I liked that I could move at my own pace and just focus on getting through my shift without being micromanaged.”

- IDI 37, age 21–24, Male, San Francisco, Cash Now, parent

Taken together, these narratives illustrate how time-related precarity stemming from unpredictable and irregular shifts, insufficient hours, and the need to juggle multiple jobs constrained economic stability, agency over time use, and the ability to plan for the future.

### 3.3. Coping with unsafe and unsupported work environments

This theme captures a dimension of employment precarity characterized by routine exposure to emotionally taxing and, at times, unsafe work environments, alongside limited institutional support. Participants described these experiences through three closely intertwined features: feeling devalued through limited recognition and blocked advancement, working in high-stress or risky settings with frequent exposure to conflict and crisis, and having limited access to training, benefits, or workplace support. Rather than describing these as separate problems, participants portrayed them as overlapping conditions that made work difficult to sustain and contributed to ongoing strain.

While a small number of participants were employed in settings that offered mentorship, professional development, or alignment with personal values, most recounted work environments where high demands were paired with minimal support or opportunity for growth. Across accounts, participants described having limited leverage to request safer conditions, additional training, or schedule stability.

#### 3.3.1. Lack of recognition and opportunities for advancement

Participants commonly described feeling overlooked or taken for granted, even when they assumed responsibilities beyond their formal roles. This sense of being undervalued was often tied to stagnant wages and the absence of clear promotion pathways. One participant working a seasonal retail job described being relied upon during emergencies without receiving tangible recognition or advancement:

“It’s like no matter what I do, I’m stuck in the same spot. I handle fights, I deal with emergencies, and I’ve even stepped in when other people didn’t show up. But there’s no recognition, no raises, no promotions. It’s just, ‘Good job, see you next shift.’ And I can’t keep doing this forever.”

- IDI 21, age18–20, Female, San Francisco, Cash Later

For participants in contingent or part-time roles, limited access to full-time work reinforced a sense of disposability and exclusion from workplace belonging. One participant with nearly a decade of retail experience described feeling peripheral and easily replaceable:

“I only work part-time. So it’s like I have a lot of free time. […] Most people—they only need you if they really need you. If somebody calls out, they're going to need you to come in. So I kind of feel a little bit like—like an extra. I don’t feel like that’s actually like—I don’t feel a part of the team.”

- IDI 14, age 21–24, Female, San Francisco, Cash Now

Only a small number of participants described workplaces where they felt recognized or saw clear opportunities for growth. One participant employed at a nonprofit contrasted this experience with prior jobs in which he felt easily replaceable, emphasizing how recognition and trust shaped his sense of contribution and sustainability at work:

“My boss was looking to do a presentation … surrounding education and transforming the way we educate minorities. And he did end up selecting me to go take that presentation with him. And it was a great personal development moment … Oftentimes … work can often just feel really unimportant and petty because it can seem as though, like, if you were to croak tomorrow, they could replace you today. It’s really a very cog-in-the-machine-ish [feeling] in some places. And so I don’t experience that feeling here … I do feel that my impact is [real] - we only have a staff of 30, so I know my impact is important.”

- IDI 5, age18–20, Male, Oakland, Cash Now

#### 3.3.2. Unsafe and emotionally distressing work environments

For many participants, experiences of devaluation were reinforced by routine exposure to unsafe or emotionally distressing working conditions. Participants employed in security guard roles, particularly in higher-risk settings such as homeless encampments or hospitals, described frequent exposure to conflict, crisis, and potential violence. These environments were often characterized by limited on-site supervision and minimal recognition of the emotional and physical demands involved. One participant described working conditions marked by constant disruption and risk, alongside an absence of managerial presence:

“Every day there was a fight between an employee and an unhoused resident, fires, or escorting out non-residents. It was never quiet, never safe. Supervisors were never present on site, so there was no recognition for what we were dealing with. We were just expected to handle it, no matter how bad things got.”

- IDI 21, age18–20, Female, San Francisco, Cash Later

#### 3.3.3. Lack of training, protections, and support

Participants also described limited access to training, protections, and managerial support as compounding these risks. Training was often described as generic or insufficient for the specific demands of their work settings, particularly in high-acuity or high-risk environments. One security guard working in a psychiatric hospital explained that training was delivered as a single standardized session across multiple hospital sites, leaving her unprepared for the distinct safety risks, forms of aggression, and physical demands she encountered in psychiatric care settings. She emphasized that the absence of site-specific preparation made it difficult to anticipate or manage routine incidents, underscoring how inadequate training functioned as a form of institutional neglect rather than protection. Participants also reported being rotated across roles, closely monitored for mistakes, and expected to absorb heavy workloads without adequate preparation or support, making work difficult to sustain over time.

Several participants further described limited accommodation for health needs, where absences or reduced capacity placed continued employment at risk. Across these accounts, devaluation, exposure to risk, and lack of institutional support operated together to produce a sense of being “stuck,” with few viable pathways out of precarious employment.

### 3.4. Exercising constrained agency

This theme synthesizes the preceding findings by examining how BEA exercised agency within the structural constraints described in Themes 3.1–3.3. Using case studies, we illustrate how participants actively made decisions about employment, caregiving, and self-investment in the context of employment instability, time-related precarity, and unsafe or unsupported work environments. Rather than representing a distinct dimension of precarity, these cases demonstrate how agency is enacted under constraints. The three cases presented were selected as illustrative examples based on narrative richness and longitudinal completeness, highlighting common patterns observed across the broader sample despite shared financial need and participation in the same guaranteed income intervention.

Brianna, Isaiah, and Andre's cases both shared patterns and divergent trajectories among Black emerging adults navigating precarious employment. All three exercised agency, but always within the boundaries of labor market exclusion, limited safety nets, and uneven institutional support. Differences in caregiving responsibilities and access to opportunity structured how far that agency could translate into stability.

Brianna leveraged guaranteed income and a nonprofit internship to achieve key developmental milestones, including a Guard Card and driver's license, temporarily expanding her employment options. However, sustaining these gains depended on continued external support. Isaiah similarly accepted a welfare-linked trainee position that enabled him to meet caregiving obligations, but offered few transferable skills or pathways to stability, while the combined demands of caregiving and employment strain undermined his health. In contrast, Andre used guaranteed income to concentrate fully on Fire Academy training. In the absence of caregiving responsibilities and with access to a vocational pathway, his efforts translated into upward mobility, albeit alongside new occupational risks and ongoing housing affordability constraints.

Together, these cases demonstrate that participants were not passive recipients of income or opportunities, but active decision-makers navigating survival, caregiving, and future planning under constraint. Yet the returns on agency were highly uneven. Brianna's progress was fragile, Isaiah's constrained by caregiving and temporary placements, and Andre's enabled by circumstances not shared by many peers. Across the sample, few participants experienced sustained improvements in employment quality over 12 months, underscoring how agency is exercised within structural limits that shape what forms of progress are possible.

### 3.5. Precarious employment and health

Across interviews, participants described a reciprocal relationship between health and precarious employment. Pre-existing physical and mental health conditions shaped participants’ ability to obtain, sustain, or advance in employment, while precarious work arrangements in turn exacerbated symptoms, limited opportunities for recovery, and interfered with access to care over time. Rather than operating as a one-directional pathway, health and employment conditions were experienced as mutually reinforcing across the study period.

#### 3.5.1. Mental health

Several participants entered the study with pre-existing mental health conditions, including anxiety, depression, or trauma-related symptoms, which shaped the kinds of jobs they could pursue and tolerate. Participants described difficulty sustaining employment in jobs with unstable schedules, high demands, or limited support, particularly when symptoms worsened. In turn, unpredictable hours, income volatility, and exposure to stressful or unsafe work environments intensified psychological distress. Participants described persistent worry when hours were cut without notice, frustration at being unable to plan ahead, and distress when progress could not be maintained from one pay period to the next. One participant with pre-existing anxiety, depression, and PTSD described how being rapidly moved across departments with little support intensified symptoms and ultimately required hospital care:

“They wanted me in the kitchen department, then domestics, then the cash register when the store opened, then back downstairs, and then back upstairs. My manager told me he was going to hire new people so I wouldn’t have to work like that, but he hired them for another department … They just kept overworking me. I already have anxiety, depression, and PTSD. […] It just seemed like every time one little thing goes wrong, they wanted to pull me aside or pull me to the office to tell me about it. But they never told me how good I was doing, even though I was on my hands and knees and literally hurting myself. I’ve got to go to the hospital because of them.”

- IDI 18, age 21–24, Female, San Francisco, Cash Now

Her account illustrates how high demands paired with low support could intensify pre-existing symptoms and, in some cases, push participants toward crisis care or job exit. Participants working in security guard positions described frequent exposure to conflict and crisis as emotionally taxing, particularly when combined with limited supervision or preparation. For others, psychological strain stemmed from routine exposure to crisis and potential violence as a normal part of the job.

“We get a lot of 5150s - people that are a harm to themselves or others. They bring a lot of different things on them like knives, pepper sprays. I had an experience where somebody brought a gun into the hospital. I had to take it from them and call the sheriffs. […] Yeah, it’s a little stressful, but it’s okay.”

- IDI 30, age 21–24, Female, Oakland, Cash Later

Even when participants minimized stress, these descriptions suggest sustained emotional labor and hypervigilance embedded in everyday work. Across accounts, mental health symptoms both shaped employment decisions and were exacerbated by precarious work conditions, contributing to cycles of instability.

#### 3.5.2. Physical health, work demands, and access to care

Participants also described how precarious employment affected physical health through long hours, multiple job holding, and limited time for rest, meals, and recovery.

“I got [anemia] after I had my son … I’m still anemic … sometimes I don’t have enough iron in what I eat … it’ll get super cold, to the point where I’m shaking.”

- IDI 4, age 18–20, Female, Oakland, Cash Now, parent

Fatigue and physical exhaustion were widely reported, particularly among those juggling shifts across multiple positions or supplementing income through informal work. Some participants described worsening chronic conditions in the context of job loss or unstable employment, while others continued working through illness due to fear of losing income or employment altogether.

Work conditions also directly interfered with access to healthcare. Participants described avoiding appointments due to fear of losing hours or shifts, as well as job loss following absences for illness or surgery.

“[I] have a chronic skin condition, and it got really bad, and I had to go into surgery. So, I don’t want to say I got fired because of that, but basically, because of me missing work so much, they gave my position up. Even though they said I could reapply. It’s basically like I got fired. You know? Like, I have to reapply to work.”

- IDI 7, age 21–24, Female, San Francisco, Cash Now

Irregular schedules, long commutes, and unpredictable hours further limited participants' ability to establish routine care, even when health insurance coverage was available. These constraints contributed to unmet health needs and delayed recovery, which in turn affected participants’ capacity to sustain employment.

#### 3.5.3. Health and employment over time

Across the 12-month study period, few participants described sustained improvements in either employment quality or health. In some cases, participants described short-term improvements in stress or stability during periods when supports were available, but these gains were difficult to maintain once supports ended. For others, ongoing employment instability contributed to cumulative strain, with health challenges limiting employment options and precarious work conditions further undermining health. Together, these accounts illustrate how health and precarious employment shaped one another over time, contributing to persistent instability during the transition to adulthood.

## 4. Discussion

This qualitative study examined how low-income BEA participating in a guaranteed income trial experienced and navigated precarious employment and how these conditions shaped their health during early labor market entry. Although links between precarious employment and poor health are well established, this study adds detail on how precarity is lived during emerging adulthood by identifying four interrelated dimensions of participants' work lives: employment instability (low wages, high turnover), time-related precarity (unpredictable schedules, scarcity of reprieve), unsafe and unsupported work environments, and constrained agency. Across accounts, unstable work arrangements structured daily routines, constrained future planning, and disrupted healthcare seeking, while unsafe or unsupported workplaces intensified physical and emotional strain. Participants’ narratives further suggest that time scarcity and constrained agency operate as integrative mechanisms connecting multiple domains of precarious employment to health during early adulthood, highlighting how precarity is shaped by life stage, place, and racialized labor market conditions, with potential implications for later health and economic stability.

### 4.1. Structural pathways to health inequity

Our findings align with prior research showing that precarious employment contributes to poorer physical and mental health through material deprivation, psychosocial stress, and exposure to unsafe conditions (Benach et al., 2014; Hajat et al., 2024; Kreshpaj et al., 2020; Lewchuk et al., 2018; Pulford et al., 2022). Participants’ accounts demonstrate that these pathways can take shape early in the life course, when individuals are entering the labor market and transitioning into adulthood. Our study builds on longitudinal evidence that early job quality is associated with long-term health and economic mobility (Amick et al., 2016; Eisenberg-Guyot et al., 2020).

The structural dimensions of precarity were experienced by participants as harmful to health and future opportunity. Employment instability (Theme 3.1) and low wages limited participants' capacity to plan for near-term goals such as education, training, caregiving arrangements, and housing stability. Many participants assembled a patchwork of multiple part-time or temporary jobs, often through staffing agencies or short-term contracts, with little opportunity for advancement. These conditions mirror national trends showing rising employment precarity among young and low wage workers (Kalleberg, 2018; Oddo et al., 2021). In our sample, such instability directly constrained participants’ capacity to pursue education or training, supporting prior work that identifies early employment quality as a critical determinant of later socioeconomic status and health (Bradley & Corwyn, 2002; Duncan et al., 2010).

Furthermore, time-related precarity (Theme 3.2) was experienced by participants as a distinct, potentially health-harming mechanism. Unpredictable and inflexible schedules disrupted rest, routines, and medical care, reflecting the temporal dimensions of precarious employment that limit control over daily life (Hajat et al., 2024). This limited control over time compounded material deprivation and stress, especially for parents balancing caregiving with multiple low-wage jobs. These findings underscore the importance of control over time within employment quality frameworks.

Finally, unsafe and unsupportive work environments (Theme 3.3), characterized by violence, inadequate training, and absence of oversight, further eroded health. Limited access to training, workplace accommodation, and on-site managerial support among temporary and precarious workers is a well-documented feature of contemporary labor markets, particularly in sectors relying on subcontracting, staffing agencies, and short-term contracts (Benach et al., 2014; Kalleberg, 2011). Our findings align with this prior work while extending it by illustrating how these training gaps are experienced during emerging adulthood and how they are shaped by structural racism and constrained by caregiving responsibilities. Several participants continued to work while sick or avoided medical care due to fear of job loss, highlighting how weak employment protections can transform short-term health issues into chronic problems.

### 4.2. Constrained agency and bidirectional health effects

Agency was central to how participants navigated structural constraint. Similar to Silva's description of working class young adults striving for stability within narrow opportunity structures (Silva, 2013), BEA in this study made deliberate decisions about employment, education, and caregiving. However, agency operated within tight “bounded” limits shaped by labor market exclusion and limited safety nets (Emirbayer & Mische, 1998). Participants exercised creativity and persistence, yet most remained in precarious or unstable work over the 12-month follow-up period. The diverging outcomes of case studies (Brianna, Isaiah, Andre) illustrate how the returns on individual effort are shaped by contextual factors like caregiving status, vocational pathways, and external supports.

The relationship between precarious employment and health was distinctly bidirectional. Participants described physical strain, fatigue, and mental distress resulting from unstable hours, low pay, and unsafe workplaces. These health challenges, in turn, limited their ability to sustain or improve employment conditions. The data illustrate how precarious employment can both produce and perpetuate health inequities, echoing prior findings that poor quality employment functions as both a determinant and a consequence of poor health across the life course (Amick et al., 2016; Benach et al., 2014; Eisenberg-Guyot et al., 2020).

### 4.3. The role of guaranteed income

Participants receiving transfers during the 12-month qualitative follow-up (Cash Now) described guaranteed income as temporarily easing financial pressure with effects varying by context. In particular, those who also accessed mentoring or training, described modest improvements in employment stability, while others used it to meet immediate needs without achieving durable gains. These patterns are consistent with research showing that income supports can buffer short term hardship but must be paired with workforce development and childcare policies to alter structural conditions (Bor et al., 2017; Lippman et al., 2023). While guaranteed income reduced stress and enabled some participants to make short term investments in education or housing, the underlying features of precarious work (e.g., low wages, unsafe environments, and unpredictable schedules) remained largely unchanged.

### 4.4. Policy implications

Our findings highlight precarious employment as a structural determinant of health that may shape early life trajectories. During emerging adulthood, instability in work and income intersects with the developmental tasks of education, caregiving responsibilities, housing stability, and employment transitions, which may amplify the consequences of poor employment quality over time. Addressing these conditions requires moving beyond income supplementation to structural investments. Policies that ensure predictable scheduling, mandatory minimum wages tied to cost-of-living, and access to safe workplaces could protect both economic and physical health. Programs and interventions that integrate income support with education and training opportunities, accessible childcare, and genuine pathways to stable employment may be especially important for promoting social and economic mobility among BEA.

### 4.5. Strengths and limitations

This study has several strengths. The longitudinal design captured changes in employment and health over time, and the diverse sample included participants with diverse gender identities, caregiving responsibilities, and city of residence, providing a range of experiences. The use of framework analysis supported systematic comparison across participants and time points. Limitations include that the interview guide was not designed specifically around all established employment quality constructs, which may have constrained discussion of some dimensions, and that agency emerged inductively rather than through targeted inquiry. As a qualitative study, our findings do not establish causal relationships but instead illuminate how participants described and interpreted connections between precarious employment and their health. Future research using longitudinal and mixed-methods designs could help to more directly assess causal pathways linking employment quality and health across the transition to adulthood.

## 5. Conclusion

This study shows how participants described precarious employment as undermining the health of BEA by generating instability, time scarcity, unsafe conditions, and constrained agency. These structural dynamics limited participants’ ability to pursue education, provide care, and access needed health services, potentially reinforcing disadvantage at a critical life stage. While guaranteed income offered temporary relief, its impact depended heavily on other support systems and ultimately was insufficient to overcome structural labor market barriers. The findings highlight that precarious employment is not merely an economic issue, but also an important social determinant of health that can compound racial inequities over time. Efforts to promote health equity must extend beyond income to include stronger workplace protections, accessible childcare, and investment in stable employment pathways to disrupt cycles of precarity and advance health equity.

## Figures and Tables

**Table 1:** Characteristics of study participants at baseline (N =36) and 12-months follow- up (N =31).

	Baseline N (%)	12-months N (%)

**Age at baseline**		
18–20	20 (55.6)	18 (58.0)
21-24	16 (44.4)	13 (41.9)
**Gender**		
Man	16 (44.4)	13 (41.9)
Woman	16 (44.4)	15 (48.4)
Transgender/Non-binary	4 (11.2)	3 (8.3)
**City of residence**		
Oakland	16 (44.4)	13 (41.9)
San Francisco	20 (55.6)	18 (58.0)
**Study arm**		
Cash now	19 (52.8)	17 (54.8)
Cash later	17 (47.2)	14 (45.2)

Note: Age categories reflect participants’ age at baseline. For consistency, participants were retained in their baseline age category at 12-month follow-up even if they aged into the next category during the study period.

## Data Availability

The datasets generated and analyzed during the current study are not publicly available due to the sensitivity of qualitative data and confidentiality agreements with study participants. De-identified excerpts may be available from the corresponding author on reasonable request and pending IRB approval.

## CASE STUDY 1: BRIANNA

### Background:

Brianna, 20, lived in Oakland with her mother, sister, and young son. After giving birth during high school, she dropped out and began navigating early adulthood as a single mother without a high school diploma.

### Baseline:

At baseline, Brianna worked part-time at a fast-food chain, a job she had held for five months. The position offered low wages, no benefits, and an erratic schedule that complicated her ability to secure childcare and manage household expenses. Although she was covered by Medi-Cal (California's Medicaid program), she reported unmet healthcare needs and had discontinued use of prescribed medication for depression and bipolar disorder. To supplement her wages, Brianna regularly donated plasma, despite having been diagnosed with anemia, illustrating the difficult health tradeoffs participants described when managing basic needs under financial strain.

### 12-Month Follow-Up:

By follow-up, Brianna had left her job after what she described as “slacking on the job,” citing stress and worsening mental health. During her first year in BEEM, she received monthly guaranteed income payments of $500, participated in financial mentoring sessions, and completed a six-month paid internship through a local nonprofit. Through this job readiness internship, she also received a Guard Card, prepared for her GED, and obtained her driver's license marking key developmental gains. These supports appeared to bolster her self-confidence and skillset. However, by the end of the 12-month period, both the guaranteed income payments and job readiness support had ended, leaving her once again unemployed and financially vulnerable. These efforts reflect sustained agency under conditions of instability, though progress remained contingent on continued external support.

## CASE STUDY 2: ISAIAH

### Background:

Isaiah, 23, lived in San Francisco with his partner, their two daughters, his mother, and younger brother. At enrollment, he had a history of temporary work and was sharing parenting responsibilities with his partner. His partner primarily managed household logistics, including enrollment in government assistance programs.

### Baseline:

Isaiah had recently begun working part-time as a public service trainee with the city parks department, a position accessed through a case worker as part of a state welfare program. Though the aim of the position was to provide job skills, Isaiah reported gaining few translatable skills and saw no clear career trajectory. However, the position did provide flexibility, which he valued for balancing his caregiving responsibilities. With the guaranteed income payments supplementing his wages, Isaiah reported being able to cover his family's basic needs and expressed optimism about his future.

### 12-Month Follow-Up:

By the end of the program, Isaiah had separated from his partner and become the primary caregiver for their children. Although his trainee contract had been extended to its maximum duration, he was left without work and had not secured new employment despite actively applying. While jobs in delivery services were available, he expressed reluctance due to scheduling rigidity, which conflicted with his childcare duties. As he explained, the stress of unstable employment compounded the demands of caregiving, leaving him exhausted and anxious about how to provide for his children. Health and employment were deeply intertwined, and poor-quality, temporary placements did little to support his long-term stability, and the emotional and physical strain of both caregiving and job loss further constrained his ability to pursue better opportunities. His choices illustrate how agency exercised in the context of caregiving and temporary placements yielded limited returns.

## CASE STUDY 3: ANDRE

### Background:

Andre, 22, grew up in the Bay Area and had recently moved into a private room in Hunter's Point to focus on training at the Fire Academy. Prior to this, he lived in a crowded multigenerational household in San Francisco with his mother, stepfather, three sisters, younger brother, and stepfather's parents. He described frequent moves between relatives' homes and was aware of the broader dynamics of gentrification in San Francisco and another Bay Area city, where he had also lived. Unlike Brianna and Isaiah, Andre did not have children.

### Baseline:

At baseline, Andre was enrolled in the Fire Academy, a demanding training program he described as tough but fulfilling. His path to firefighting began with work as an EMT, which exposed him to emergency response and reinforced his interest in “giving back to the community” and “helping support the city.” Before training, he worked at an afterschool program in Bayview. Although he had once attended an out-of-state university on an academic scholarship, financial strain and social isolation led him to return to the Bay Area. Attempts to resume school at a community college were derailed when he was charged out-of-state tuition. Reflecting on these experiences, he noted a loss of motivation for formal education. Andre supported himself through the BEEM guaranteed income payments, which he used primarily for rent. His savings had been depleted by family emergencies, and he was eager to rebuild financial independence. While he had not regularly accessed healthcare since childhood, he recently completed a required physical exam for the Fire Academy. He had not sought mental health services, though he described close relationships with family, particularly his mother and grandmother, as key sources of emotional support.

### 12-Month Follow-Up:

Andre had successfully completed the 20-week Fire Academy and was working full-time as a firefighter. His new role involved 24-h shifts, often followed by 48 h off, and he described the intensity of responding to multiple fires in one shift. The work was physically and mentally demanding, but he expressed pride in his ability to manage the responsibility. His financial stability improved with a steady paycheck, which allowed him to save money, purchase a car, and begin building credit. He described adopting disciplined budgeting practices and saw these as part of “adulting” and becoming more self-sufficient. Andre remained in a modest shared housing arrangement but aspired to eventually rent a studio apartment on his own. He weighed this goal carefully against cost and sustainability, cautious not to make a move that would jeopardize his progress. He also acknowledged the occupational health risks of firefighting and expressed intentions to establish regular medical care moving forward. His trajectory highlights how agency can translate into upward mobility when constraints are fewer, even as new risks emerge.
